# Perceiving greater commitment increases selfishness among disagreeable people

**DOI:** 10.1371/journal.pone.0303693

**Published:** 2024-06-03

**Authors:** Raini N. Sizemore, Levi R. Baker

**Affiliations:** Department of Psychology, The University of North Carolina at Greensboro, Greensboro, North Carolina, United States of America; Polytechnic Institute of Coimbra: Instituto Politecnico de Coimbra, PORTUGAL

## Abstract

Perceiving that a partner is highly committed tends to benefit close relationships. However, there may be relational drawbacks to perceiving high commitment. In particular, given that high commitment may signal that a partner is unlikely to leave the relationship, perceiving that a partner is highly committed might lead people low in agreeableness to feel comfortable behaving more selfishly toward that partner. One correlational study consisting of a highly diverse sample of individuals (*n* = 307), one observational study of newlywed couples (*n* = 202), and one experiment with undergraduate couples (*n* = 252) examined whether the implications of perceived partner commitment for selfish behaviors depend on agreeableness. Results demonstrated that perceiving high commitment resulted in more selfish behavior among disagreeable participants (Studies 1–3), but less selfish behavior among agreeable participants (Studies 1 and 3). Together, these results suggest that signaling commitment to disagreeable partners may backfire in romantic relationships.

## Introduction

It is not uncommon for partners’ personal goals to conflict with one another [1], and when such goal conflicts occur, people must decide whether to prioritize their own goals or their partner’s [2]. For example, after discovering that his partner, Lucy, would rather watch a movie instead of the hockey game that he prefers, Ricky must decide whether to prioritize his own well-being by watching the game or Lucy’s well-being by watching the movie. The decisions that result from these interdependence dilemmas have important implications [3]; although being willing to behave selflessly tends to improve relationship quality [4], such selfless acts can also harm individual well-being [5], especially when they fail to improve relationship quality [6].

Although selfishness is central to numerous interpersonal processes such as altruism [7], equity [8], trust [9], and power [10], scholars have only recently directed their attention to the construct itself [see 11, 12]. Selfishness is defined as behaving in a manner that benefits the self at the expense of others [11]. In the context of romantic relationships, selfishly-motivated people tend to provide lower levels of support [13] and be less responsive [14] to their romantic partner’s needs. However, research on selfishness in romantic relationships is scarce and questions about what shapes selfish behavior in romantic relationships remain unanswered.

The extent to which people believe their partners are committed to the relationship (i.e., *perceived partner commitment*; [15]) may influence these decisions; however, theory and research make competing predictions about the direction of this influence. On the one hand, people may behave *less* selfishly toward partners who they perceive are highly committed because they may reason that those committed partners would be more likely to reciprocate such selfless behavior compared to less committed partners [16, 17]. On the other hand, given that partners are more likely to minimize transgressions [18] and less likely to end a relationship [see 19] to the extent that they are committed to that relationship, people may behave *more* selfishly toward partners who they perceive are highly committed because they may expect fewer harmful consequences from behaving selfishly toward committed partners compared to less committed partners.

Given these competing theoretical predictions, the current research seeks to identify whether perceived partner commitment increases or decreases intimates’ selfishness. The remainder of this introduction is comprised of four parts. The first section reviews theory and research that suggests that people should behave *less* selfishly to the extent that they perceive their partner is committed to their relationship. In contrast, the second section reviews theory and research that suggests that people should behave *more* selfishly to the extent that they perceive their partner is committed to their relationship. The third section attempts to reconcile these conflicting arguments by describing theoretical and empirical evidence that suggests that whether perceived partner commitment increases or decreases selfishness depends on intimates’ agreeableness. The final section describes three studies that test this possibility.

### Perceived partner commitment may decrease selfish behaviors

Perceived partner commitment refers to people’s beliefs about the extent to which their partner desires for their relationship to persist [15, 20]. Perceiving that a partner is committed to the relationship tends to benefit romantic relationships in numerous ways. For example, perceiving that a partner is committed to the relationship tends to assuage concerns that the partner is romantically interested in others [21] and thus increases trust [1] and reduces negative emotions (e.g., jealousy; [22]) and behaviors (e.g., confrontation; [23]). Further, people who believe their partners are highly committed tend to report greater relationship satisfaction [24], commitment [25], quality [26], and stability [20], compared to those who doubt their partners’ commitment.

Further, there are reasons why perceiving a partner is committed may also benefit relationships by decreasing selfish behaviors. First, people tend to be more satisfied with partners who they perceive are highly committed (vs. relatively less committed; [20]) and relationship satisfaction tends to decrease selfish behavior [1]. Second, given that partners’ commitment reflects their desire to maintain a lasting relationship, people should expect longer-lasting relationships with partners they are perceive are highly committed (vs. less committed), and people tend to sacrifice more for their partners when they believe that the relationship is likely to persist [4]. Finally, people tend to sacrifice more for their partners if they believe those partners are similarly willing to sacrifice for them [27] and highly committed partners tend to sacrifice more than less committed partners [1, 28]. In sum, people may behave less selfishly toward partners they perceive are highly committed, compared to those they perceive are less committed, because they should be more satisfied with those partners, expect longer relationships with those partners, and expect those partners to reciprocate such selfless acts.

Previous literature also provides indirect evidence that perceived partner commitment may decrease selfish behaviors. For example, research on trust suggests that people tend to sacrifice more for partners that they trust (vs. those they do not; [29]) and judgments of trust are shaped by perceptions of partners’ commitment [1]. Similarly, theory [7] and research [28] on reciprocal altruism suggest that people sacrifice more for others whom they believe would reciprocate such sacrificial acts compared to those they believe would not sacrifice for them. Given that commitment is positively associated with the willingness to sacrifice [1], individuals who perceive their partners are more committed, and thus more willing to sacrifice for them, should be more willing to forgo their own interests compared to individuals who perceive their partners to be less committed.

### Perceived partner commitment may increase selfish behaviors

Nevertheless, other theoretical perspectives suggest that perceived partner commitment may instead *increase* selfish behaviors in romantic relationships because people who perceive that their partners are highly committed should anticipate fewer harmful interpersonal consequences from their selfish behavior compared to those who believe their partners are less committed. In particular, because highly committed people are more motivated to maintain their relationships, they tend to view their partners in a more positive light than do less committed people [30]. Accordingly, individuals tend to overlook or minimize the severity of their partners’ undesirable behavior (e.g., selfishness) to the extent that they are committed to their relationship with those partners [18]. Thus, people who perceive that their partners are highly committed should be more likely to anticipate that those partners would overlook, and thus be more likely to engage in, selfish acts, compared to people who perceive their partners are less committed.

Several lines of research also indirectly support this argument. For example, research on aggression in romantic relationships suggests that people who are highly committed are more likely to experience dating violence than are less committed people [31, 32]. One reason why they may be more likely to experience aggression is that their partners may perceive them to be relatively unlikely to leave their relationships in response to their hostile behavior. Indeed, people who are constrained to their relationships and thus are unable to leave those relationships tend to experience higher rates of aggression than do those who are more able to leave [33]. Although committed individuals are still able to leave their relationships, high commitment may signal that they are similarly likely to tolerate selfish behavior. Research outside of close relationships also suggests that people may be more likely to behave selfishly when they anticipate few harmful consequences. For example, studies that employ monetary dictator games that require participants to allocate money between themselves and other players have revealed that people allocate resources more selfishly when they are anonymous, and thus are free of consequences or retribution, compared to when their identity is known [34].

### The moderating role of agreeableness

Given that perceived partner commitment might decrease selfish behavior by increasing affection and trust toward a partner yet increase selfish behavior by increasing the exploitability of that partner, whether perceived partner commitment increases or decreases selfish behavior may depend on whether the motivation to preserve the relationship by maximizing the partner’s well-being is greater than the motivation to maximize one’s own well-being. Specifically, we hypothesized that agreeableness would moderate the relationship between perceived partner commitment and selfishness. Agreeableness is a personality trait that reflects active concern for others’ welfare [35]. Although people high in agreeableness tend to be trusting, tolerant, and cooperative, people low in agreeableness tend to be cynical, greedy, and antagonistic [35] and motivated by self-interests [36], even at the expense of others [37]. For someone who is high in agreeableness and thus is motivated to preserve the relationship by maximizing their partner’s well-being, perceiving that a partner is committed signals that their partner is trustworthy and caring [1] and should reassure that person that their selfless behavior will not be exploited by their partner. Thus, perceived partner commitment may decrease selfish behavior among those high in agreeableness. However, for people who are low in agreeableness and thus are motivated to maximize their own well-being, perceiving that a partner is committed should provide that person with the opportunity to meet their goal of maximizing their self-interests [36] because their partners’ commitment can be a signal of relationship stability [38] and thus suggests that their partner may not hold them accountable for selfish acts. Thus, perceived partner commitment may increase selfish behavior among those low in agreeableness.

### Overview of the current studies

Given that theory provides competing predictions about the direction in which perceived partner commitment may shape selfishness, the goal of the current research was to examine whether agreeableness determines whether perceived partner commitment increases or decreases selfishness. Study 1 was a correlational study that examined whether perceived partner commitment and agreeableness interact to predict selfishness using two measures of selfishness (i.e., questionnaire, welfare trade-off task) with a diverse sample of undergraduates and crowd-sourced participants. Study 2 was an observational study of newlyweds that assessed their tendency to behave in a selfish manner during problem-solving discussions. Finally, Study 3 was an experiment that manipulated perceived partner commitment and subsequently assessed self-reported and observed acts of selfishness among undergraduate couples. Other than Study 2, which was a part of broader study that contained numerous measures that are irrelevant for the current hypothesis, we report all manipulations, measures, and exclusions in these studies.

Research conducted by Lemay and Dobush [39] provides initial support for these predictions. In particular, in a study of 53 heterosexual couples, they demonstrated that participants’ agreeableness marginally (*p* < .10) moderated the association between their perceptions of their partner’s commitment and observed acts of hostility toward those partners. The current research builds on these findings in three important ways. First, as noted, the interaction of agreeableness and perceived partner commitment failed to reach traditional levels of significance. Although it is possible that this is a truly null effect, it is also possible that this effect was simply underpowered due to the modest sample size. As such, each of the three current studies relied on samples that were roughly twice as large (or greater), and thus provide a stronger test of these predictions. Second, the current studies are diverse in regard to (a) design (i.e., cross-sectional, experimental), (b) type of sample (i.e., online crowd-sourced, undergraduate participant pools, community samples), and (c) demographics (i.e., ethnicity, age, relationship length, gender). As such, the current research provides a more externally valid test of these predictions. Similarly, the experimental manipulation in Study 3 increases confidence in their internal validity. Finally, the current research examines a broader outcome than previous research. Whereas previous research examined the implications of perceived partner commitment for acts of hostility, which can reflect selfish motives, the current research examined a variety of selfish behaviors (i.e., observed demands, self-reported acts of selfishness, allocation of resources). Because of these methodological differences, the current research will greatly increase confidence in the effect originally reported by Lemay and Dobush [39].

## Study 1

Study 1 examined the implications of perceived partner commitment for a broad range of selfish behaviors. Specifically, Study 1 relied on a diverse sample that consisted of both university students and Amazon MTurk participants to ensure that we observed highly-variable levels of commitment. In addition, we used two measures of selfishness: one that captured participants’ self-reported tendencies to engage in various types of selfish behaviors and one that served as a behavioral measure of selfishness. Preregistration information, all materials, and the dataset for Study 1 can be found at https://osf.io/twyr4/?view_only=4b5db9fef016495a8168b103b081af60.

### Methods

#### Participants

Participants were 413 individuals in romantic relationships. Following our preregistered criteria, 106 participants were excluded because they failed two or more attention checks. Thus, the final sample consisted of 307 participants. An a priori power analysis anticipating a medium effect-size (r^2^ = .16) indicated that a minimum of 44 participants was needed to have sufficient power (.80, two-tail, α = .05) to detect the interaction of perceived partner commitment and agreeableness. This anticipated effect size was based on the results from Study 2, which were obtained prior to conducting Studies 1 and 3, but are presented after Study 1 to present a more cohesive narrative. Given that it is suggested that approximately four times the number of participants is required to have sufficient power to detect simple effects [see 40], we made the a priori decision to recruit a minimum of 176 participants. To increase variability and external validity, participants were recruited from two locations: Amazon Mechanical Turk (*n* = 153; 49.8%) and the undergraduate participant pool at the authors’ university (*n* = 154; 50.2%). Participants were eligible for the study if they (a) had been involved in a romantic relationship for a minimum of three months, (b) were at least 18 years old, and (c) spoke English. All participants were recruited in the spring of 2021.

Participants recruited through MTurk (77 females, 75 males, 1 other) were 37.3 years of age (*SD* = 10.2 years), on average. One hundred and twenty-seven (83.0%) participants identified as heterosexual, 15 (9.8%) identified as bisexual, 3 (2.0%) identified as lesbian, gay, or homosexual, and 8 (5.2%) did not report sexual orientation. Ninety-nine (64.7%) participants identified as Caucasian, 30 (19.6%) identified as African American, 6 (3.9%) identified as Asian, 6 (3.9%) identified as Hispanic, 1 (0.7%) identified as American Indian/Alaska Native, 3 (2.0%) identified with two or more ethnicities, and 8 did not report ethnicity (5.2%).

Participants recruited through the undergraduate participant pool (121 females, 31 males, 2 did not report gender) were 20.4 years of age (*SD =* 4.03 years), on average. One hundred and thirteen (73.4%) participants identified as heterosexual, 24 (15.6%) identified as bisexual, 7 (4.5%) identified as lesbian, gay, or homosexual, 4 (2.6%) identified as another sexual orientation, and 6 (3.8%) did not report sexual orientation. Fifty-seven (37.0%) participants identified as Caucasian, 42 (27.3%) identified as African American, 23 (14.9%) identified as Hispanic, 16 (10.4%) identified as Asian, 12 (7.8%) identified with two or more ethnicities, 1 (0.6%) identified as another ethnicity, and 3 (1.9%) did not report ethnicity.

#### Procedure

Ethics statement: This study was approved by the IRB at the University of North Carolina at Greensboro (IRB #: 21–0278). Written informed consent was obtained before data collection. Participation was anonymous and participants cannot be identified.

After enrolling in the study via either MTurk or the undergraduate participant pool, all participants received a link to the study, where they completed all tasks on Qualtrics. After providing consent, participants completed self-report measures that assessed their perceptions of their partners’ commitment, their own agreeableness, and their tendency to engage in a variety of selfish behaviors in their relationship. Participants then completed a welfare trade-off task that required participants to decide whether to prioritize their own interests or the interests of their partner. Participants who were recruited from MTurk were compensated two dollars; participants who were recruited from the undergraduate participant pool were compensated with partial course credit.

#### Materials

*Perceived partner commitment*. Participants completed a version of the commitment subscale of the Investment Model Scale [41] that was modified to assess their perceptions of their partner’s commitment. This scale consisted of seven items (e.g., “My partner wants our relationship to last for a very long time”) that participants indicated their agreement with on a 9-point scale (0 = *Do not agree at all* to 8 = *Agree completely*). Internal consistency was high (α = .85).

*Agreeableness*. Participants completed the 20-item Agreeableness scale from the International Personality Item Pool [42]. This scale asks participants to indicate their agreement with items assessing agreeableness (e.g., “I sympathize with others’ feelings”) on a 5-point scale (1 = *Very inaccurate* to 5 = *Very accurate*). Internal consistency was acceptable (α = .70).

*Self-reported selfishness*. Participants completed the Selfishness Questionnaire [43] that was modified to address selfishness towards a partner. This scale consisted of 18 items (e.g., “Now and again, I’ve manipulated my partner to gain an advantage”) that participants indicated their agreement with on a 3-point scale (1 = *Disagree*, 2 *= Neither agree nor disagree*, 3 = *Agree*). Internal consistency was high (α = .93).

*Welfare trade-off task*. Participants also completed a welfare trade-off task [see 44] to assess selfishness. This task presents participants with 60 scenarios in which they have to decide whether to benefit either themselves or their partners. Participants completed one of two versions of this task, depending on their living situation. Participants who reported that they did not live with their partners completed the traditional monetary version, which gives participants the option to give varying amounts of money either to themselves or their partners (e.g., “Would you rather receive $55 or have your partner receive $49?”). Given that this decision would be inconsequential for participants who share finances, and given that participants who cohabitate often share finances [see 45], participants who cohabitate completed a modified version of this task that gave them the option to assign various minutes of household chores to either themselves or their partners (e.g., “Would you rather do 55 minutes of chores or have your partner do 49 minutes of chores?”). The values for the partner-directed choices were anchored in six sets of ten choices (anchors set at 45, 49, 63, 72, 94, and 101; either in dollar amounts or minutes of chores) and the values for the participant-directed choices systematically varied to create ten choices for each set. We followed the instructions of Kirkpatrick and colleagues [44] to score this task. First, for each choice, we calculated the ratio of the amount that person could take from their partner to the amount they could give to their partner. Next, within each of the six sets, we identified the point at which participants switched from benefiting their partner to benefitting themselves. Specifically, for each participant, within each set, we calculated the average of the ratio of the smallest amount they were *willing* to take and the ratio of the largest amount they were *unwilling* to take. For example, if the least a participant was willing to take within a set was 65 dollars (instead of giving their partner 63 dollars) and the most they were unwilling to take within that set was 57 dollars (to give their partner 63 dollars), we would calculate the average (i.e., 0.97) of the ratio of both choices (i.e., 1.03, 0.90). Finally, we calculated the average of participants’ scores on each of the six sets. The decisions of participants in the chores version of the task were reverse-coded to be equivalent to those in the monetary version. These scores were standardized and reverse-coded so that, for all participants, higher scores signify more selfish behavior.

#### Alternative moderators

In addition to agreeableness, which we predicted would moderate the effects of perceived partner commitment, we also assessed honesty-humility and altruism and conducted exploratory analyses to examine whether they similarly moderate the effects of perceived partner commitment. Results from these exploratory analyses were largely nonsignificant and inconsistent across studies and outcomes. As such, details about these alternative moderators can be found in the S1 File.

*Potential confounding variables*. To increase confidence that any obtained associations between perceived partner commitment and selfishness were not the result of their shared associations with other confounding variables, we assessed and controlled for several variables in supplemental analyses. First, given that people’s own relationship commitment tends to not only partially shape perceptions of their partner’s commitment [25, 46], but also decrease selfish behavior [47, for review, see 3], we assessed participants’ own relationship commitment with the commitment subscale of the Investment Model Scale [41] (α = .86). Second, we assessed several demographic characteristics—specifically participants’ age, sex, ethnicity, sexual orientation, and relationship length—that might account for any obtained results.

### Results

#### Descriptive statistics and preliminary analyses

Descriptive statistics and bivariate correlations are reported in Table 1. Although selfishness scores were around the midpoint of the scale, suggesting that participants were moderately selfish on average, there was considerable variability in their responses and some participants reported relatively high levels of selfishness. Men and women did not differ in agreeableness, *t*(302) = 0.85, *p* = .396, *d* = 0.10, 95% CI = [-.13, .34], or welfare trade-off scores, *t*(299) = -1.38, *p* = .169, *d* = -0.17, 95% CI = [-.41, .07]. However, women reported higher perceived partner commitment scores than men, *t*(302) = -2.53, *p* = .012, *d* = -0.30, 95% CI = [-.54, -.07]. Conversely, men reported higher selfishness questionnaire scores than women, *t*(302) = 3.58, *p* < .001, *d* = 0.43, 95% CI = [.19, .67].

**Table 1:** Descriptive statistics and correlations among variables in Study 1.

	Variable	1	2	3	4	*M*	*SD*
(1)	Perceived Partner Commitment		-.21**	-.11	.03	7.65	1.40
(2)	Agreeableness	-.22*		-.16*	-.21**	3.25	0.41
(3)	Selfishness Questionnaire	-.15	-.11		.35**	1.55	0.44
(4)	Welfare Tradeoff	-.13	-.03	.38**		0.07	0.96
	*M*	7.20	3.30	1.75	-0.10		
	*SD*	1.62	0.43	0.52	1.07		

*Note*. Descriptive statistics and correlations are presented above the diagonal for women and below the diagonal for men.

* *p* < .05.

** *p* < .01.

#### Does agreeableness moderate the association between perceived partner commitment and selfishness?

**Fig 1:**
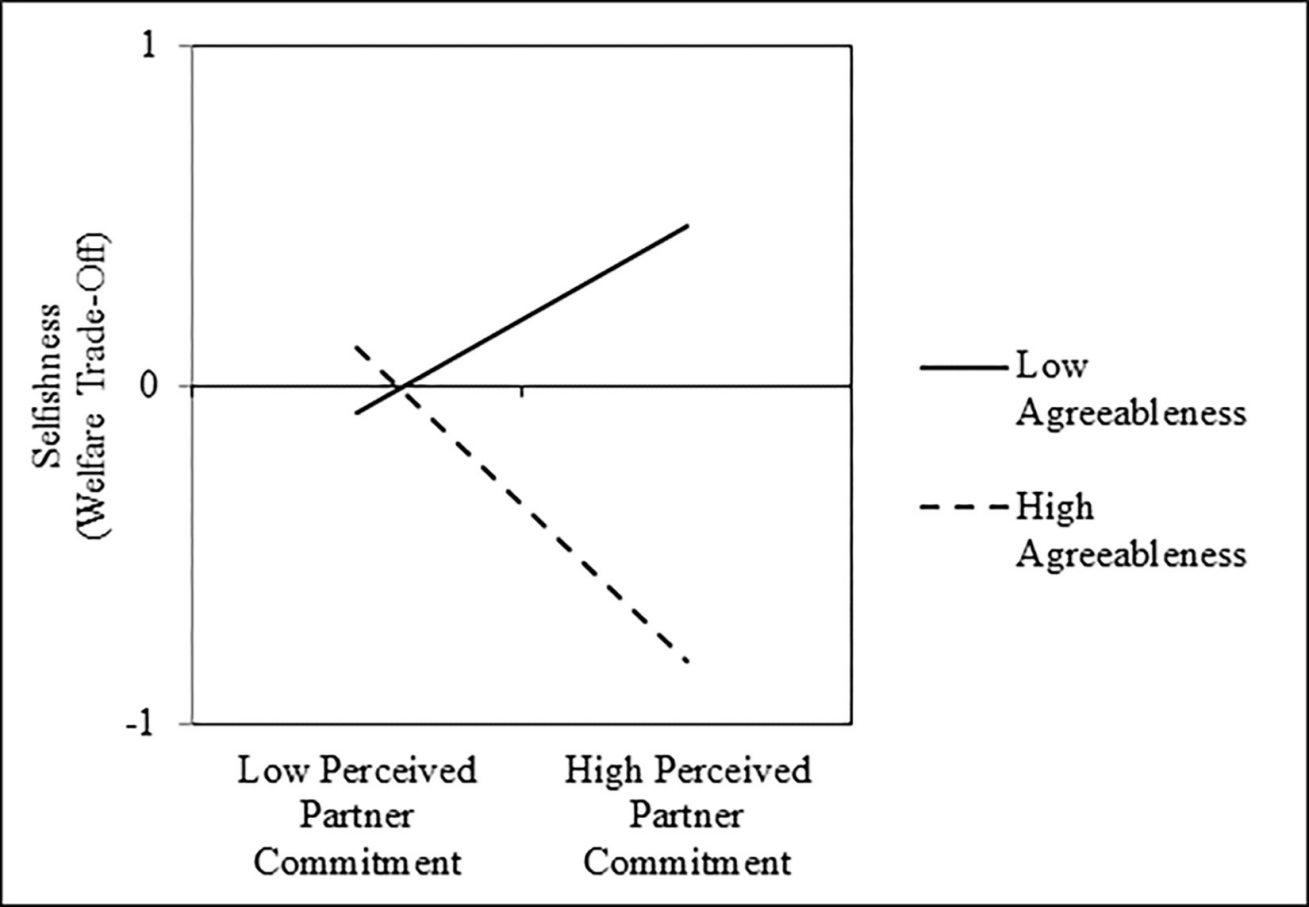
Interactive effects of perceived partner commitment and agreeableness on selflessness, as measured by the welfare trade-off task, in Study 1.

To address the primary hypothesis, we first regressed participants’ scores on the *welfare trade-off task* onto mean-centered perceived partner commitment scores, mean-centered agreeableness scores, and their interaction. Results of these analyses are presented in the left columns of Table 2. As shown, perceived partner commitment was not associated with selfish behavior on the welfare trade-off task, on average; however, this null main effect was qualified by a significant Perceived Partner Commitment × Agreeableness interaction (Fig 1). Tests of the simple slopes revealed that perceived partner commitment was negatively associated with scores on the welfare tradeoff task among people who were one standard deviation above the mean in agreeableness, *b* = -0.31, *SE* = 0.06, *t*(300) = -5.64, *p* < .001, *r* = -.31, 95% CI = [-.41, -.21], but positively associated with scores on the welfare trade-off task among people who were one standard deviation below the mean in agreeableness, *b* = 0.19, *SE* = 0.05, *t*(300) = 3.61, *p* < .001, *r* = .20, 95% CI = [.09, .31]. Further, two supplemental analyses revealed that this interaction remained significant after controlling for participants’ own relationship commitment, *b* = -0.58, *SE* = 0.09, *t*(299) = -6.23, *p* < .001, *r* = -.34, 95% CI = [-.44, -.24], and a set of participants’ demographic characteristics that included their age, a dummy-code for their sex (0 = *male*, 1 = *female*), a dummy-code for their ethnicity (0 = *White*, 1 = *non-White*), a dummy-code for their sexual orientation (0 = *heterosexual*, 1 = *non-heterosexual*), and relationship length in months, *b* = -0.62, *SE* = 0.09, *t*(282) = -6.77, *p* < .001, *r* = -.37, 95% CI = [-.46, -.27]. Finally, this interaction was not moderated by sample (i.e., undergraduate vs. MTurk), *b* = -0.01, *SE* = 0.11, *t*(296) = -0.06, *p* = .957, *r* = -.00, 95% CI = [-.11, .11], or task version (i.e., money vs. chores), *b* = -0.01, *SE* = 0.11, *t*(296) = -0.11, *p* = .915, *r* = -.01, 95% CI = [-.12, .10].

**Table 2:** Effects of perceived partner commitment, agreeableness, and their interaction on selfishness in Study 1.

	Welfare Trade-Off				Selfishness Questionnaire			
Measure	*b*	*t*	*r*	*p*	*b*	*t*	*r*	*p*
PPC	-0.06	-1.69	-.10	.092	-0.07	-4.11	-.23	< .001
Agreeableness	-0.66	-4.77	-.27	< .001	-0.35	-5.47	-.30	< .001
PPC × Agreeableness	-0.60	-6.51	-.35	< .001	-0.32	-7.48	-.39	< .001

*Note*. PPC = Perceived Partner Commitment. For the welfare trade-off task, *df* = 300. For the selfishness questionnaire, *df* = 303.

**Fig 2:**
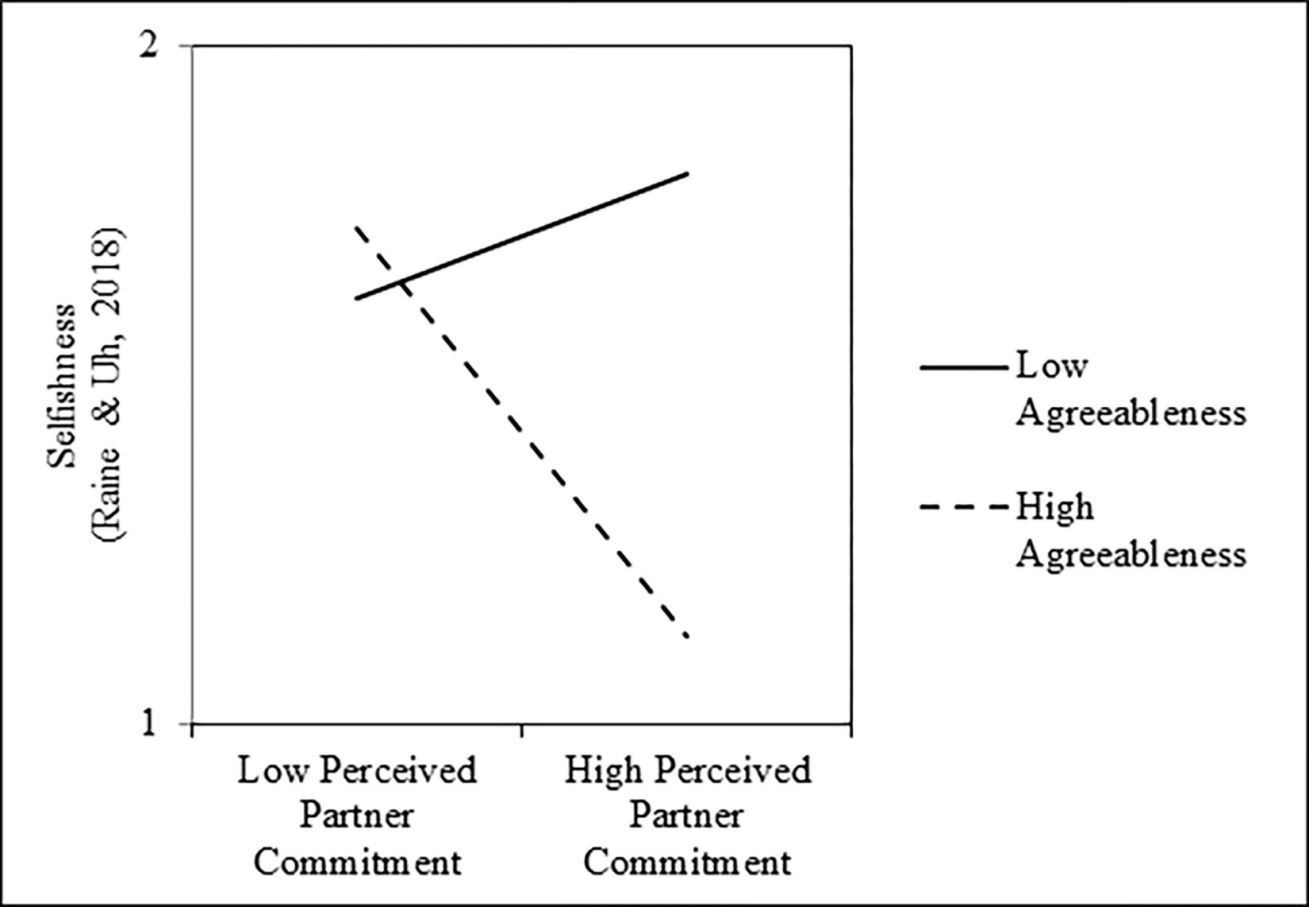
Interactive effects of perceived partner commitment and agreeableness on selfishness, as measured by the selfishness questionnaire, in Study 1.

Next, we examined whether a similar pattern would emerge with participants’ *self-reported selfishness*. Specifically, we regressed participants’ scores on the selfishness questionnaire onto mean-centered perceived partner commitment scores, mean-centered agreeableness scores, and their interaction. Results of these analyses are presented in the right columns of Table 2. As shown, perceived partner commitment was associated with less selfish behavior, on average; however, this main effect was qualified by a significant Perceived Partner Commitment × Agreeableness interaction (Fig 2). Tests of the simple slopes revealed that perceived partner commitment was associated with less selfish behavior among people who were one standard deviation above the mean in agreeableness, *b* = -0.20, *SE* = 0.03, *t*(303) = -8.00, *p* < .001, *r* = -.42, 95% CI = [-.51, -.32], but associated with greater selfish behavior among people who were one standard deviation below the mean in agreeableness, *b* = 0.06, *SE* = 0.02, *t*(303) = 2.59, *p* = .010, *r* = .15, 95% CI = [.04, .26]. Further, two supplemental analyses revealed that this interaction remained significant after controlling for participants’ own relationship commitment, *b* = -0.30, *SE* = 0.04, *t*(302) = -7.11, *p* < .001, *r* = -.38, 95% CI = [-.47, -.28], and a set of participants’ demographic characteristics that included their age, a dummy-code for their sex (0 = *male*, 1 = *female*), a dummy-code for their ethnicity (0 = *White*, 1 = *non-White*), a dummy-code for their sexual orientation (0 = *heterosexual*, 1 = *non-heterosexual*), and relationship length in months, *b* = -0.27, *SE* = 0.04, *t*(285) = -6.43, *p* < .001, *r* = -.36, 95% CI = [-.45, -.26]. Finally, this interaction was not moderated by sample, *b* = 0.02, *SE* = 0.05, *t*(299) = 0.39, *p* = .696, *r* = .02, 95% CI = [-.09, .13].

## Discussion

Study 1 provided preliminary evidence that perceived partner commitment interacts with agreeableness to predict selfishness using both a self-report measure of previous behavior and a measure of hypothetical behavior. Specifically, perceived partner commitment was associated with greater selfishness among intimates who were low in agreeableness but associated with less selfishness among intimates who were high in agreeableness. Further, this pattern of results replicated across two types of assessments: one that assessed their previous selfish behaviors and one that assessed their selfish responses to hypothetical trade-off scenarios. Nevertheless, one important limitation of Study 1 was that it relied on participants’ reports of their behavior, which may be inaccurate. Study 2 addressed this issue by observing a naturally occurring selfish behavior during problem-solving discussions.

## Study 2

Data for Study 2 came from a broader study of newlyweds that examined the extent to which participants would engage in a naturally occurring, specific type of selfish behavior: demanding their spouse change their behavior to meet their own preferences. Participants first completed measures of agreeableness and perceived partner commitment and then engaged in problem-solving discussions that were later coded for demanding behavior. Study 2 was conducted prior to the development of the current hypotheses and thus not preregistered. Further, because of the potential for people to access their partner’s data if those data are publicly available [48], we did not obtain consent from participants to make their data publicly available; however, these data and a full list of measures from the broader study are available to researchers who wish to confirm the results upon request.

### Methods

#### Participants

Participants were 101 newlywed couples participating in an ongoing broader study of marriage. This sample size was the maximum number of couples we had the funds to recruit. Of the 101 couples, 93 were in mixed-sex relationships and 8 were in same-sex (7 female-female, 1 male-male) relationships. On average, participants were 32.35 years old (*SD* = 8.71). One hundred and twenty-nine (64%) participants identified as White or Caucasian, 55 (27%) identified as Black or African American, four (2%) identified as Asian or Asian-American, three (2%) identified as Latino/a, one (1%) identified as American Indian or Alaska Native, one (1%) identified as Native Hawaiian or other Pacific Islander, and the remaining 9 (5%) identified as two or more ethnicities. One hundred and seventy-eight (88%) participants identified as heterosexual, thirteen (6%) identified as gay or lesbian, ten (5%) identified as bisexual, and one did not report their sexual orientation. Couples were recruited through invitations sent to couples who had applied for marriage licenses in the county. Couples were screened in a telephone interview to ensure they (a) had been married for less than three months, (b) were at least 18 years old, and (c) spoke English. All participants were recruited from the fall of 2015 to the fall of 2016.

#### Procedure

Ethics statement: This study was approved by the IRB at the University of North Carolina at Greensboro (IRB #: 15–0368). Written informed consent was obtained before data collection. Because participants needed to be contacted for follow-up assessments that were unrelated to the current hypotheses, participation was confidential but not anonymous.

Participants were first emailed a link to the Baseline survey, which was hosted on Qualtrics. This survey included a consent form approved by the IRB at UNCG, self-report measures that included measures of agreeableness and perceived partner commitment, and instructions to complete all questionnaires independently of their spouse. Next, couples scheduled and attended a laboratory session where they participated in two problem-solving discussions designed to assess how they resolve problems in their relationship. Before each discussion, each spouse identified a problem that affected their relationship or an aspect of their relationship that they would like to change. After identifying topics for the discussions, both spouses participated in two, eight-minute videotaped discussions in which they were left alone to “work toward some resolution or agreement” for each problem. The order of the discussions was determined at random. If both partners happened to choose the same topic, that topic was discussed first, followed by a second topic chosen by the spouse who was randomly determined to be discussed second.

#### Materials

*Perceived partner commitment*. Participants completed the modified version of the commitment subscale of the Investment Model Scale [41] used in Study 1. Internal consistency was acceptable (α = .68).

*Agreeableness*. Participants completed the 10-item Agreeableness scale from the International Personality Item Pool [42]. This scale asks participants to indicate their agreement with items assessing agreeableness (e.g., “I sympathize with others’ feelings”, “I take time out for others”) on a 5-point scale (1 = *Very inaccurate* to 5 = *Very accurate*). Internal consistency was high (α = .82).

*Demanding behavior*. Couples’ problem-solving behaviors were coded from videotapes of their problem-solving discussions. Coders used a global, interval coding system to quantify participants’ demanding behavior. In particular, coders assigned a code for each two-minute segment of each eight-minute conversation that indicated both the frequency and severity in which each participant “demanded or pressured their partner to change their behavior” using a scale from 1 (*Did not do this at all*) to 7 (*Severe and frequent demands*). Coders assigned codes for each two-minute interval, rather than providing one code for the entire conversation, to reduce the possibility that primacy and recency effects [see 48] would bias their coding. The eight codes for each participant (four codes for each conversation) were averaged together to form an index of how much each person tended to demand changes across the two conversations. Approximately 75% of the conversations were coded by a second researcher. Intraclass correlation coefficients indicated that the coders were reliable (ICC = .76).

*Potential confounding variables*. Similar to Study 1, we assessed and controlled for several variables in supplemental analyses. Specifically, we again assessed participants’ own relationship commitment with the commitment subscale of the Investment Model Scale [41] (α = .59) and participants’ demographic characteristics—specifically their age, sex, ethnicity, sexual orientation, and relationship length.

### Results

#### Descriptive statistics and preliminary analyses

Descriptive statistics and bivariate correlations appear in Table 3. Although demanding scores were below the midpoint of the scale, it is worth noting that these scores represent the average demands across the four short segments and it would be unexpected for participants to engage in high levels of demanding behavior across the entire conversation. Indeed, the level of demands that participants engaged in were similar to other oppositional behaviors reported in similar studies [49, 50]. Men and women did not differ in perceived partner commitment, *t*(200) = -0.42, *p* = .677, *d* = -0.06, 95% CI = [-.34, .22]. However, women’s self-reported agreeableness was higher than men’s self-reported agreeableness, *t*(200) = -2.38, *p* = .018, *d* = -0.34, 95% CI = [-.61, -.06]. Similarly, women exhibited more demanding behavior than men, *t*(200) = -2.67, *p* = .008, *d* = -0.38, 95% CI = [-.66, -.10].

**Table 3:** Descriptive statistics and correlations among variables in Study 2.

Variable	1	2	3	*M*	*SD*
(1) Perceived Partner Commitment	**.24** ^*^	.01	.02	8.53	.85
(2) Agreeableness	.12	**-.06**	-.04	4.01	.61
(3) Demands	.13	.13	**.02**	1.68	.54
*M*	8.48	3.81	1.50		
*SD*	.96	.59	.38		

*Note*. Descriptive statistics and correlations are presented above the diagonal for women and below the diagonal for men; correlations between spouses appear on the diagonal in bold.

* *p* < .05.

#### Does agreeableness moderate the association between perceived partner commitment and demands?

**Fig 3:**
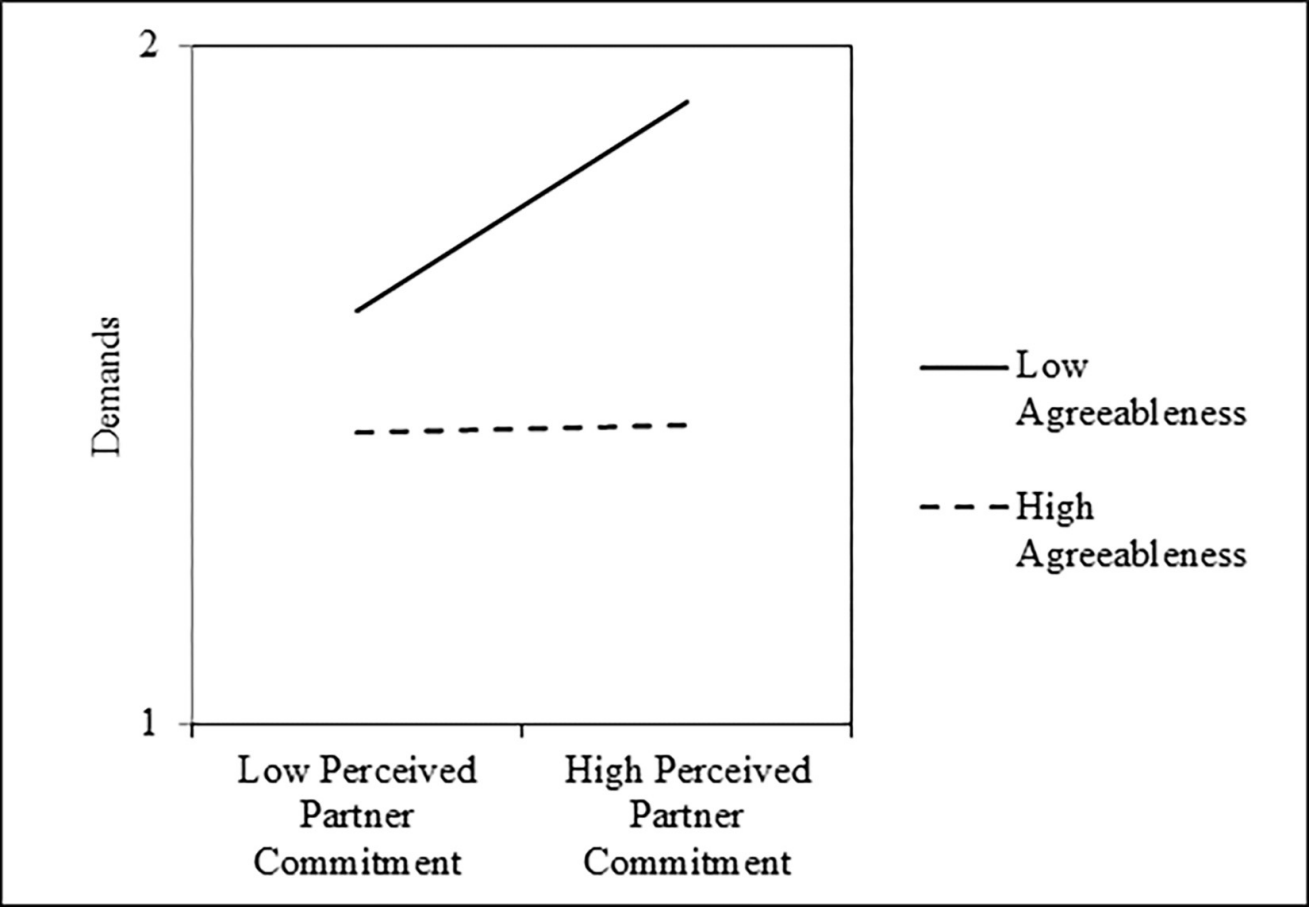
Interactive effects of perceived partner commitment and agreeableness on demands during problem-solving discussions in Study 2.

To address whether the implications of intimates’ perceptions of their partners’ commitment for the extent to which they demanded behavioral changes from their partners depended on their own levels of agreeableness, we estimated a two-level model using the HLM 7.03 computer program [51]. In the first level of the model, participants’ demand scores were regressed onto their mean-centered perceived partner commitment scores, mean-centered agreeableness scores, and their interaction. The non-independence of couples’ data was controlled in the second level of the model, which allowed for a randomly varying intercept. Results are presented in Table 4. As shown, perceived partner commitment was significantly associated with less demanding behavior, on average; however, this main effect was qualified by a significant Perceived Partner Commitment × Agreeableness interaction (Fig 3). Tests of the simple slopes revealed that perceived partner commitment was associated with greater demands among intimates who were one standard deviation below the mean in agreeableness, *b* = 0.02, *SE* = 0.01, *t*(97) = 4.67, *p* < .001, *r* = .43, 95% CI = [.31, .54], but not among intimates who were one standard deviation above the mean in agreeableness, *b* = 0.00, *SE* = 0.00, *t*(97) = 0.21, *p* = .837, *r* = .02, 95% CI = [-.12, .16]. Further, two supplemental analyses revealed that this interaction remained significant after controlling for participants’ own relationship commitment, *b* = -0.00, *SE* = 0.00, *t*(96) = -4.52, *p* < .001, *r* = -.42, 95% CI = [-.53, -.30], and a set of participants’ demographic characteristics that included their age, a dummy-code for their sex (0 = *male*, 1 = *female*), a dummy-code for their ethnicity (0 = *White*, 1 = *non-White*), a dummy-code for their sexual orientation (0 = *heterosexual*, 1 = *non-heterosexual*), and relationship length in months, *b* = -00, *SE* = 0.00, *t*(91) = -4.52, *p* < .001, *r* = -.43, 95% CI = [-.54, -.31]. Finally, subsequent analyses indicated this interaction was not further moderated by partners’ sex, *b* = -0.00, *SE* = 0.00, *t*(93) = -0.74, *p* = .461, *r* = -.08, 95% CI = [-.22, .06], and remained significant when estimating an actor–partner interdependence model (APIM; [52]) that controlled for their partners’ agreeableness, perceived partner commitment, and demands, *b* = -0.00, *SE* = 0.00, *t*(93) = -3.43, *p* = .001, *r* = -.34, 95% CI = [-.46, -.21].

**Table 4:** Effects of perceived partner commitment, agreeableness, and their interaction on demands during problem-solving discussions in Study 2.

	Demands			
Measure	*b*	*t*	*r*	*p*
PPC	0.01	3.32	.32	.001
Agreeableness	-0.03	-4.44	-.41	< .001
PPC × Agreeableness	-0.00	-4.24	-.40	< .001

*Note*. PPC = Perceived Partner Commitment. *df* = 97.

### Discussion

Study 2 provided further support that perceived partner commitment interacts with agreeableness to predict a specific form of selfish behavior: demanding that a partner change. Specifically, perceived partner commitment was associated with greater demanding behavior among intimates who were low in agreeableness but not among intimates who were high in agreeableness. Nevertheless, both Studies 1 and 2 were limited by their correlational designs, which limited the causal conclusions that could be drawn from them. Study 3 addressed this by experimentally manipulating perceived partner commitment.

## Study 3

Study 3 sought to provide experimental evidence for the relationship between perceived partner commitment, agreeableness, and selfish behaviors. Specifically, we manipulated whether participants would perceive their partners to be high or low in commitment. We also used two measures of selfishness: a self-report of selfish behaviors and a behavioral measure of selfishness that required participants to choose the volume of a disruptive noise blast for themselves and their partners. Preregistration information and all materials for Study 3 can be found here: https://osf.io/t9bcv/?view_only=13eb7be2f741477bac6638ca15fd6457. Because of the potential for people to access their partner’s data if those data are publicly available [see 48], we did not obtain consent from participants to make their data publicly available; however, these data are available to researchers who wish to confirm the results upon request.

### Methods

#### Participants

Participants were 252 college students (126 couples). Following our preregistered criteria, three participants were excluded because they failed two or more attention checks. Thus, the final sample consisted of 249 participants (141 females, 108 males). An a priori power analysis to determine sample size indicated that, for a multiple regression analysis with three predictors, a medium effect (r^2^ = .15 [see 53]), an alpha of .05, and a power of .80, the sample size needed to include at least 55 participants. However, given that we anticipated a cross-over interaction, roughly four times the number of participants was needed to conduct sufficiently powered simple effects tests [see 40], suggesting that we needed at least 220 participants (110 couples). Due to a technical error, exact age and relationship length were not recorded. However, eligible participants were (a) at least 18 years old and (b) in a romantic relationship for a minimum of three months. Participants were recruited from the undergraduate participant pool at the authors’ university. All participants were recruited from the fall of 2021 to the spring of 2022.

Two hundred (80.3%) participants identified as exclusively dating, 27 (10.8%) identified as casually dating, 14 (5.6%) identified as married, and 8 (3.2%) identified as engaged. Ninety-four participants (37.8%) identified as White/Caucasian, 76 (30.5%) identified as Black/African American, 32 (12.9%) identified as Hispanic/Latino/a, 18 (7.2%) identified as Asian, 1 (0.4%) identified as American Indian/Alaska Native, 6 (2.4%) identified as another ethnicity not listed, and 22 (8.8%) identified as two or more ethnicities.

#### Procedure

Ethics statement: This study was approved by the IRB at the University of North Carolina at Greensboro (IRB #: FY22-6). Written informed consent was obtained before data collection. Participation was anonymous and participants cannot be identified.

Participants signed both themselves and their partner up for the study through the undergraduate participant pool. Upon arriving at the laboratory, couples were taken to separate rooms where they individually completed all aspects of the study. Participants first provided consent and completed the agreeableness questionnaire.

Participants then completed an evaluative priming task that was used to manipulate perceived partner commitment and was not actually scored [see 50, 54]. This task required participants to categorize words as either commitment-related or neutral by pressing a key on the keyboard after being primed with either their own name, their partner’s name, or a random name. After completing this task, a researcher informed participants that the task ostensibly measured their automatic feelings of commitment toward their partner. All participants were then told that they scored slightly above average (65^th^ percentile), which indicated that they were moderately committed to their partner. Participants were also told that their partners completed the same task. To manipulate perceived partner commitment, participants were randomly assigned to be told that their partner scored either above (91^st^ percentile; *n* = 137) or below (31^st^ percentile; *n* = 112) average on their evaluative priming task, indicating that their partner was either relatively high or low in commitment, respectively. Participants then completed a perspective-taking questionnaire that contained a single item that served as a manipulation check of their perceptions of their partners’ commitment (i.e., “How much does your partner care about your relationship?”) and a selfishness questionnaire.

Following this, participants were told they would participate in an ostensibly unrelated study that involved testing their cognitive abilities. First, participants completed a Stroop task [55] as a filler task. Second, participants were instructed that they would complete a memory task that would require them to memorize words with noise in the background. Participants were told that the study required testing participants at varying levels of noise. To ensure that participants were aware of the range of volumes they could hear, participants listened to samples of the noise at the lowest (i.e., 0), middle (i.e., 50), and loudest (i.e., 100) possible volumes. Next, participants were informed that they could select the volume that they would hear throughout the remainder of the task, ranging from 0 to 100; however, participants were also told that, to ensure adequate variability, their partners would hear the exact opposite of their selection. For example, if they selected a relatively loud noise (e.g., 75), their partners would hear a relatively quiet noise (e.g., 25), and vice versa. The research assistant then left the room and allowed the participant to make their choice on a sliding scale. Scores on this task were reversed so that higher scores indicated greater selfishness. After making the choice, participants were debriefed and given partial course credit for their participation.

#### Materials

*Selfishness*. In addition to the noise blast task, participants completed the modified version of the Selfishness Questionnaire [43] described in Study 1. Internal consistency was high (α = .81).

*Agreeableness*. To assess agreeableness, participants completed the Agreeableness scale [42] described in Study 1. Internal consistency was high (α = .78).

*Alternative moderators*. In addition to agreeableness, which we predicted would moderate the effects of perceived partner commitment, we also assessed honesty-humility and the Dark Triad and conducted exploratory analyses to examine whether they similarly moderate the effects of perceived partner commitment. Results from these exploratory analyses were largely nonsignificant and inconsistent across studies and outcomes. As such, details about these alternative moderators can be found in the S1 File.

*Potential confounding variables*. Similar to Studies 1 and 2, we assessed and controlled for participants’ demographic characteristics—specifically their sex and ethnicity—in supplemental analyses.

### Results

#### Descriptive statistics and preliminary analyses

Descriptive statistics and bivariate correlations are reported in Table 5. Although participants selected volumes on the noise blast task that were slightly below the midpoint of the scale, suggesting that, on average, they selected a slightly louder volume for themselves than their partners, there was considerable variability in their responses and many participants selected higher louder volumes for their partners than themselves, indicating greater selfishness. Men and women did not differ significantly on self-reported selfishness, *t*(247) = -0.41, *p* = .343, *d* = -0.05, 95% CI = [-.30, .20]. Women selected significantly greater volumes on the noise blast task, suggesting more selfishness, than did men, *t*(247) = -5.09, *p* < .001, *d* = -0.65, 95% CI = [-.91, -.39]. Finally, participants reported that their partners were more committed in the high perceived partner commitment condition (*M =* 6.68, *SD* = 0.67) than in the low perceived partner commitment condition (*M* = 6.21, *SD* = 1.16), *t*(247) = -4.03, *p* < .001, *d* = -0.51, 95% CI = [-.77, -.26], suggesting that the manipulation of perceived partner commitment was effective.

**Table 5:** Descriptive statistics and correlations among variables in Study 3.

	Variable	1	2	3	*M*	*SD*
(1)	Agreeableness	**.14**	-.28**	.09	3.89	0.42
(2)	Selfishness Questionnaire	-.41**	**.04**	.28**	1.27	0.28
(3)	Noise Blast Volume Choice	-.03	.16	**.04**	46.63	22.39
	*M*	3.82	1.26	33.78		
	*SD*	0.43	0.24	23.36		

*Note*. Descriptive statistics and correlations are presented above the diagonal for women and below the diagonal for men; correlations between partners appear on the diagonal in bold.

* *p* < .05.

** *p* < .01.

#### Does agreeableness moderate the association between perceived partner commitment and selfishness?

To address the primary hypothesis, we estimated two models. The first model examined the implications of perceived partner commitment for the *noise blast task* by estimating a two-level model using the HLM 7.03 computer program [51] that regressed participants’ volume choice onto condition (-1 = low commitment condition, 1 = high commitment condition), mean-centered agreeableness scores, and their interaction. The non-independence of couples’ data was controlled in the second level of the model, which allowed for a randomly varying intercept.

**Fig 4:**
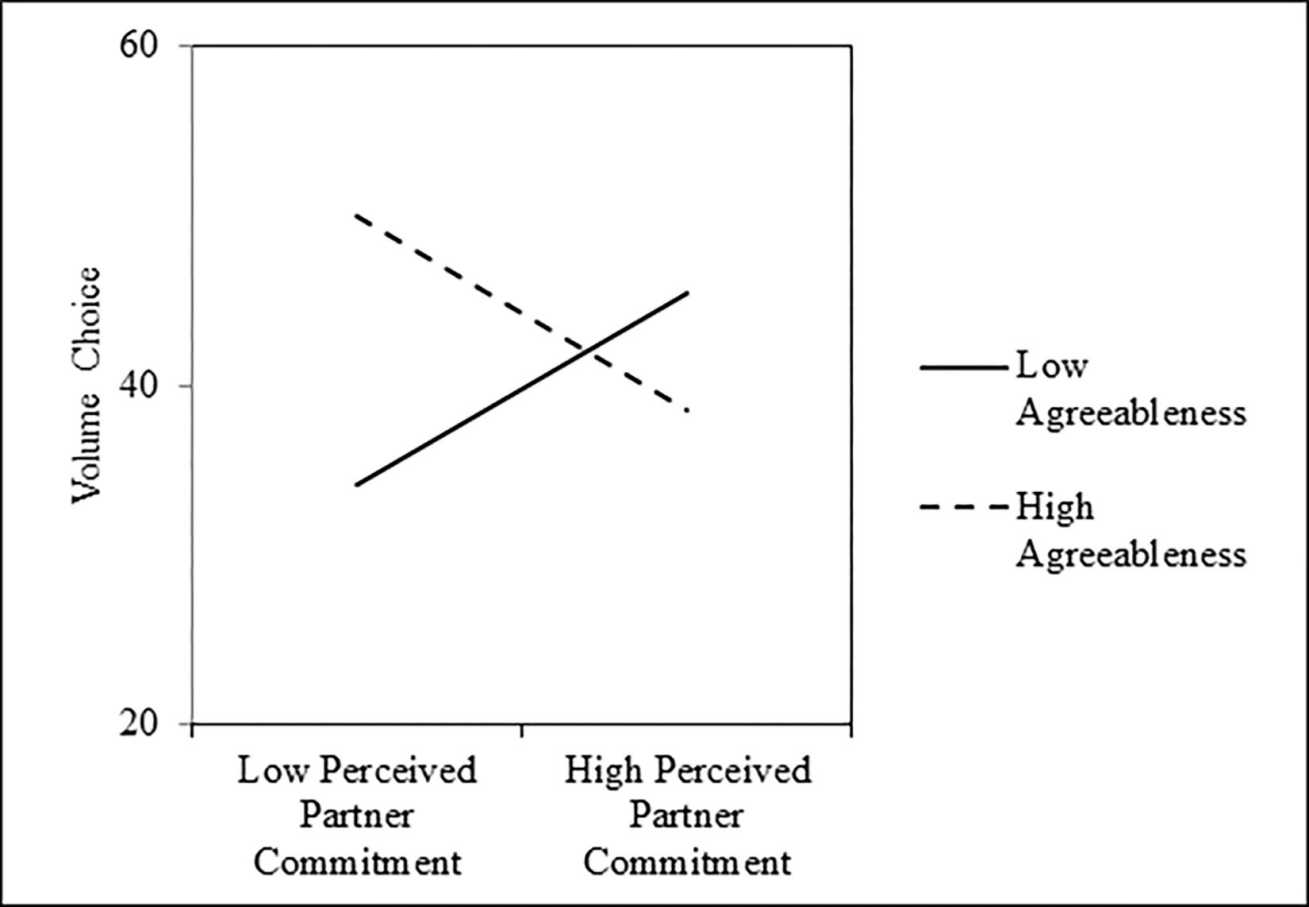
Interactive effects of perceived partner commitment and agreeableness on selfishness, as measured by the noise blast task, in Study 3.

Results of these analyses are presented in the left columns of Table 6. As shown, perceived partner commitment was not associated with the volume that participants chose, on average; however, this null main effect was qualified by a significant PPC Condition × Agreeableness interaction (Fig 4). In particular, tests of the simple slopes revealed that perceived partner commitment condition was significantly negatively associated with volume choice, which indicates less selfish behavior, among people who were one standard deviation above the mean in agreeableness, *b* = -5.69, *SE* = 2.17, *t*(120) = -2.62, *p* = .010, *r* = -.23, 95% CI = [-.34, -.11], but significantly positively associated with volume choice, which indicates more selfish behavior, among people who were one standard deviation below the mean in agreeableness, *b* = 5.64, *SE* = 2.15, *t*(120) = -2.62, *p* = .010, *r* = .23, 95% CI = [.11, .34]. Further, this interaction remained significant when controlling for a dummy-code for participants’ sex (0 = *male*, 1 = *female*) and ethnicity (0 = *White*, 1 = *non-White*), *b* = -13.16, *SE* = 3.52, *t*(118) = -3.74, *p* < .001, *r* = -.33, 95% CI = [-.21, -.44], and when estimating an actor–partner interdependence model (APIM; [52]) that controlled for their partners’ agreeableness, *b* = -13.65, *SE* = 3.64, *t*(119) = -3.75, *p* < .001, *r* = -.33, 95% CI = [-.21, -.44].

**Table 6:** Effects of perceived partner commitment condition, agreeableness, and their interaction on selfishness in Study 3.

	Volume Choice (Noise Blast Task)				Selfishness Questionnaire			
Measure	*b*	*t*	*r*	*p*	*b*	*t*	*r*	*p*
PPC Condition	-0.03	-0.02	-.00	.986	-0.01	-0.35	-.03	.728
Agreeableness	5.30	1.40	.13	.164	-0.19	-4.46	-.38	< .001
PPC × Agreeableness	-13.38	-3.60	-.31	< .001	-0.14	-3.16	-.28	.002

*Note*. PPC = Perceived Partner Commitment. For the noise blast task, *df* = 120. For the selfishness questionnaire, *df* = 120.

**Fig 5:**
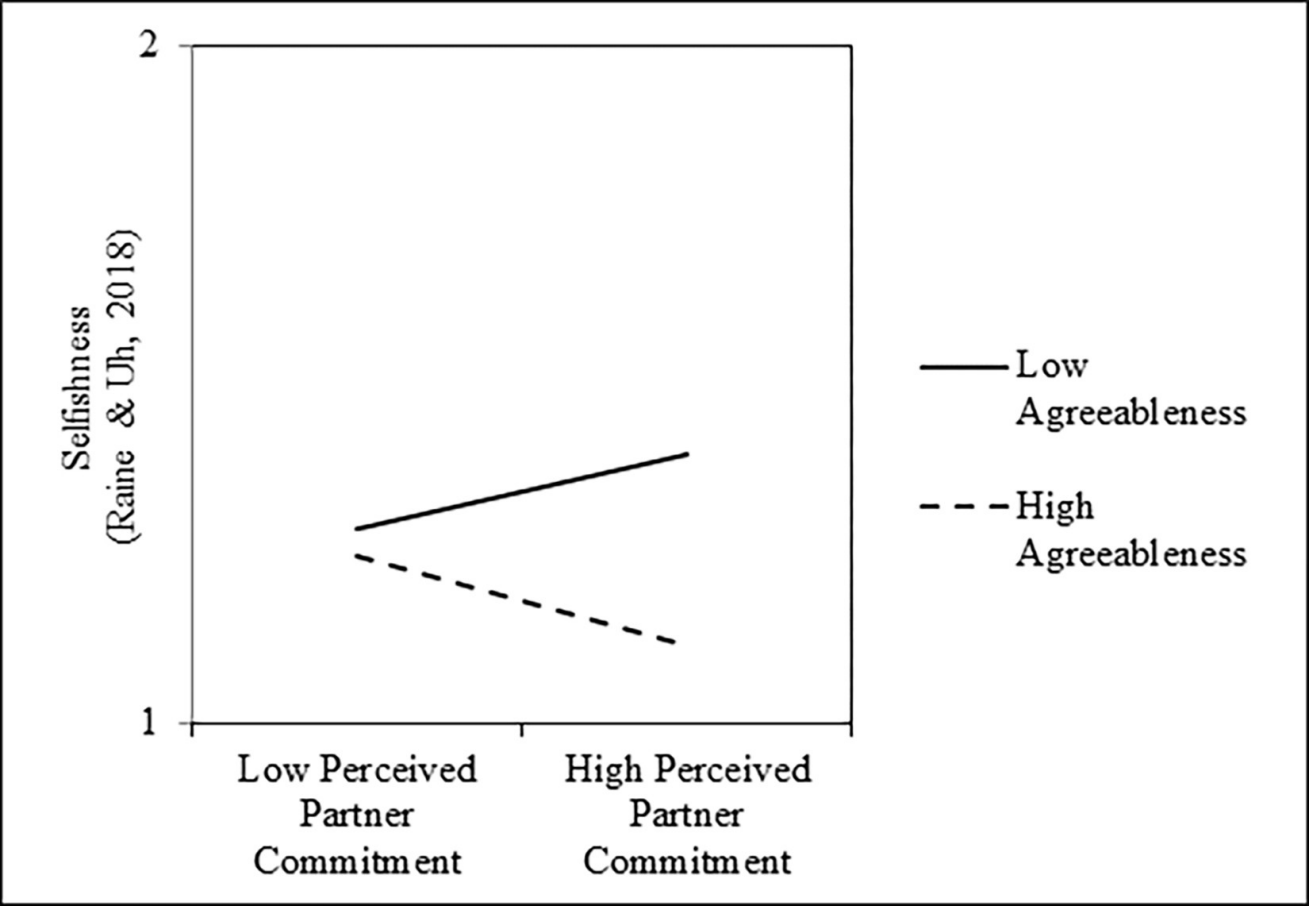
Interactive effects of perceived partner commitment and agreeableness on selfishness, as measured by the selfishness questionnaire, in Study 3.

The second model examined the implications of perceived partner commitment for participants’ *self-reported selfishness* by regressing participants’ scores on the selfishness questionnaire onto condition, mean-centered agreeableness scores, and their interaction. Results of these analyses are presented in the right columns of Table 6. As shown, perceived partner commitment was not associated with participants’ selfishness questionnaire scores, on average; however, this null main effect was qualified by a significant PPC Condition × Agreeableness interaction (Fig 5). In particular, tests of the simple slopes revealed that perceived partner commitment condition was significantly associated with less self-reported selfishness among people who were high in agreeableness, *b* = -0.06, *SE* = 0.02, *t*(120) = -3.29, *p* < .001, *r* = -.29, 95% CI = [-.40, -.17], but only marginally significantly associated with more self-reported selfishness among people who were low in agreeableness, *b* = 0.05, *SE* = 0.03, *t*(120) = 1.83, *p* = .069, *r* = .16, 95% CI = [.04, .28]. Further, this interaction remained significant when controlling for a dummy-code for participants’ sex (0 = *male*, 1 = *female*) and ethnicity (0 = *White*, 1 = *non-White*), *b* = -0.14, *SE* = 0.04, *t*(118) = -3.10, *p* = .002, *r* = -.27, 95% CI = [-.38, -.15], and when estimating an actor–partner interdependence model (APIM; [52]) that controlled for their partners’ agreeableness, *b* = 0.05, *SE* = 0.3, *t*(119) = -3.30, *p* = .001, *r* = -.30, 95% CI = [-.41, -.18].

## General discussion

How does perceiving that a romantic partner is committed influence selfishness? Previous research can be used to suggest conflicting arguments. On the one hand, people may behave less selfishly if they perceive their partners to be highly committed because they should be more satisfied with those partners, expect longer relationships with those partners, and expect those partners to reciprocate such selfless acts compared to people who perceive their partners are less committed. On the other hand, people may behave more selfishly if they perceive their partners are highly committed because high commitment may signal that a partner is less likely to end the relationship as a result of their selfish behavior. Prior research [39] provides initial evidence that agreeableness might determine whether perceived partner commitment increases or decreases selfish behavior in romantic relationships.

In three studies, we replicated and extended these findings. In Study 1, participants’ perceptions of their partners’ commitment were associated with greater self-reported tendencies to behave selfishly and greater selfishness in a welfare trade-off task among intimates who were low in agreeableness but less selfishness among intimates who were high in agreeableness. In Study 2, newlyweds’ perceptions of their spouses’ commitment were associated with greater demanding behaviors among those low in agreeableness, but not among those high in agreeableness. Finally, in Study 3, leading intimates to believe that their partners were highly committed increased self-reported and observed selfishness among intimates low in agreeableness, but decreased selfishness among those high in agreeableness. As noted, these studies were sufficiently powered and diverse in regard to (a) design (i.e., cross-sectional, experimental), (b) type of sample (i.e., online crowd-sourced, undergraduate participant pools, community samples), (c) demographics (i.e., ethnicity, age, relationship length, gender), and (d) types of selfish behaviors assessed (i.e., observed demands, self-reported acts of selfishness, allocation of resources and annoying stimuli), increasing confidence in the internal, external, construct, and statistical validity of this pattern of results. Importantly, controlling for participants’ own relationship commitment in Studies 1–2 did not affect the pattern of results, reducing the likelihood that these results emerged simply because participants’ own commitment biased both their perceptions of their partners’ commitment and their own selfish behavior.

### Implications and future directions

These findings have important theoretical implications and provide several directions for future research. First, these studies highlight a potential drawback of perceiving high commitment. Although people tend to perceive their relationships more positively when they believe their partners are committed [56] and thus engage in more prosocial ways [57], the current research revealed that commitment may be exploited by certain partners. Specifically, we show that disagreeable people, compared to agreeable people, may be more willing to take advantage of a partner to the extent that they believe their partner is committed. Thus, perceiving that a partner is committed may be harmful to some relationships, particularly those that include a disagreeable person. This mirrors previous work highlighting the negative implications of disagreeableness [37, 58] and adds to a growing body of literature that demonstrates the importance of agreeableness for romantic relationships [54, 59]. However, the current research does not imply that disagreeable people will always act selfishly or agreeable people will always act selflessly. In particular, these data suggest that a disagreeable person may choose to act selflessly if they believe their partner may punish them for selfish behavior. Similarly, an agreeable person may choose to act selfishly if they believe they may be taken advantage of by a partner who will not reciprocate selfless behavior (i.e., an uncommitted partner). These results may be particularly informative to people entering into new relationships. Although it may be advantageous to enter into a relatively uncommitted relationship with someone who is low in agreeableness, entering into a highly committed relationship with that same person may be costly. Future research may explore other situations in which signaling commitment may backfire.

Second, these studies contribute to a growing body of literature surrounding selfishness, specifically in close relationships [60, 61]. Despite ample research highlighting the role of selfishness in general social settings [for review, see 11], there is a considerable lack of research focused on selfishness in romantic relationships. Nevertheless, the idea that intimates should avoid acting in a selfish manner is central to many perspectives on relationship maintenance [3, 7, 8]. For example, Rusbult and colleagues [3] observed that people tend to prioritize partners’ interests more, and their own interests less, as they become more committed to those partners and argue that this transformation of motivation helps sustain satisfying relationships. These ideas are also consistent with evolutionary perspectives [7] that suggest that people often make sacrifices to strengthen social bonds with others who may provide valuable benefits. Indeed, there is evidence that people avoiding selfish behavior helps people maintain relationships. For example, partners tend to be more satisfied with [5], committed to [62], and persist in relationships longer with [4] people who behave in a less (vs. more) selfish manner.

Nevertheless, there may be times when it appropriate for intimates to behave in a more selfish manner. For example, it may be adaptive for people who consistently sacrifice for their partners and/or who are exploited by those partners to occasionally prioritize their own self-interests. In these cases, not only will they personally benefit from the occasional selfish act, it may also improve the quality of their relationships by minimizing inequity between partners. Indeed, intimates tend to be happier with their relationships to the extent that neither partner benefits more than the other [8]. Similarly, some selfish behaviors—like demanding a partner change their problematic behavior—may improve relationship quality by reducing the severity of conflicts or problems [see 63]. For example, asking a romantic partner to stop contacting their amorous ex-partner may prevent conflicts by reducing jealousy and the threat of infidelity. Similarly, demanding that a spouse reduce their reckless gambling may increase the couple’s financial stability. Thus, behaviors that benefit one partner may, at times, ultimately also benefit their relationship.

Furthermore, the extent of selflessness that is appropriate or expected may differ across relationships. Although the current research focused on romantic relationships, which are often expected to be highly communal and characterized by less selfish behavior [64, 65], people often do not have those same expectations of romantic relationships that are short-term, non-exclusive, and/or primarily sexual [66, 67]. People similarly tend to expect more selfishness from their friends, coworkers, classmates, neighbors, and acquaintances, compared to their committed romantic partners [64, 65]. Given that people are expected to prioritize their self-interests more in these types of relationships, the harmful interpersonal consequences of selfishness may be mitigated in these relationships. Indeed, how people evaluate prosocial behavior often depends on whether or not the behavior exceeded expectations [see 68]. Future research may benefit by examining the role of selfishness in different relationships, specifically those in which selfless behavior may not be as normative.

Accordingly, the implications of the current studies may depend on the developmental context of the relationship. Participants in Study 2, for example, were newlywed couples who had recently publicly expressed their relationship commitment to one another; thus, these participants should have relatively few doubts about their partner’s commitment. Further, married couples tend to be highly interdependent due to typically sharing finances, cohabiting, increased cognitive interdependence (e.g., overlap in self and partner schemas), and shared social networks [69]. In relationships with high interdependence, people tend to expect relatively unselfish behavior from their partners because selfish behavior has the potential to greatly disrupt their well-being [68]. In contrast, participants in Studies 1 and 3 were university students, many of whom were casually dating their partners and thus may question their partner’s commitment more. These types of relationships are typical of emerging adults, who often prioritize their education and career advancement over establishing a highly committed and interdependent relationship [70]. As such, they may tolerate more selfish behavior from their partners because they may instead expect that they will both focus more on their individual development. As such, the interpersonal implications of selfish behaviors may be less harmful among emerging adults. Future research should address whether the implications of selfish behaviors differ across different developmental stages, as well as examine whether the results from the current studies replicate among other developmental stages (e.g., older adults).

Third, these studies join a growing body of literature that highlights the importance of evaluating partner perceptions [20, 39, 71, 72]. Previous work has shown that perceptions tend to guide behavior [see 73] and the current studies provide evidence that partner perceptions influence relational behavior. Although research has addressed several different types of perceptions in romantic relationships (e.g., responsiveness [71]; support [74]), the current research joins a growing body of work focusing on perceived partner commitment [20, 21, 39, 75]. Importantly, many factors influence the development of such perceptions, such as attachment [76], depression [77], perceived similarity [78], and even one’s own relationship commitment [25, 46]. As such, these perceptions about romantic partners may not be accurate [see 79], and thus future research may also explore the implications of accuracy for partner perceptions. Indeed, perceptions of partners’ thoughts and behaviors often have unique effects beyond, and are sometimes more important than, what those partners *actually* think or how they act [80]. For example, people’s behavior is influenced more by their perceptions of their partner’s commitment than their partner’s actual commitment [39].

### Strengths and limitations

The current research has several strengths. First, a similar pattern of results was obtained across multiple diverse samples that included college students and MTurk individuals (Study 1), a community sample of newlyweds (Study 2), and undergraduate couples (Study 3), thus increasing confidence in these phenomena. Second, we similarly used various designs (i.e., correlational, experimental), assessments (i.e., observational, behavioral, self-report), and operationalizations of selfishness (i.e., demanding changes from the partner, prioritizing self-interests), thus further increasing confidence in these results.

Despite these strengths, several limitations should be addressed. First, there may be concerns with relying on self-reports of selfishness. In particular, past research suggests that people tend to underestimate their undesirable behaviors [see 81]. Although the rates of selfishness that participants in these samples reported were similar to those reported in other studies [43], it is likely that people generally underreport the extent of their selfishness. Nevertheless, confidence in these results is bolstered by the behavioral measures in all three studies that revealed a similar pattern of results. Still, it is worth noting that the behavioral measure of selfishness in Study 1 may not have high ecological validity. Specifically, the welfare trade-off task used in Study 1 was hypothetical and thus participants were aware that their choices would not affect their relationship or their partner, which may have influenced how they responded. Future research may benefit from using more direct measures of selfishness that provide higher ecological validity. Finally, due to a technical error when programming Study 3, important demographic information relating to the makeup of the sample is missing (i.e., exact age, relationship length).

### Conclusion

What determines whether people prioritize their own or their partner’s well-being when faced with goal conflict dilemmas? The current studies suggest that a unique combination of perceived partner commitment and agreeableness may influence these decisions. Specifically, the current studies revealed that, contrary to previous literature [1, 21], perceiving that a partner is highly committed may have drawbacks for some individuals. More specifically, high perceived partner commitment may induce more selfishness in partners who are less agreeable, but less selfishness in partners who are more agreeable. Overall, these results highlight the unique effect that agreeableness has on selfishness and suggest that there may be negative consequences to perceiving that a partner is committed to a romantic relationship.

## Supporting information

S1 File (DOCX)
