# Supplementary material for: Perceiving greater commitment increases selfishness among disagreeable people
Source: PLoS One. 2024 Jun 3;19(6):e0303693. doi: 10.1371/journal.pone.0303693 (PMC11146695; doi:10.1371/journal.pone.0303693)
Supplement: S1 File — (DOCX) [file pone.0303693.s001.docx]

Online Supplemental Material

Perceiving greater commitment increases selfishness among disagreeable people

Raini N. Sizemore^1^, Levi R. Baker^*1^

^1^ Department of Psychology, The University of North Carolina at Greensboro, Greensboro, North Carolina, United States of America

* Corresponding author

Email: levirbaker@gmail.com (LRB)

**Study 1**

**Method**

**Materials**

**Honesty-Humility.** To rule out other potential moderators, participants completed the honesty-humility subscale of the HEXACO Personality Inventory [1], which consisted of 16 items (e.g., “I wouldn’t want people to treat me as though I were superior to them”) that participants rated their agreement with on a 5-point scale (1 = *Strongly disagree* to 5 = *Strongly agree*). This was scored by averaging all responses to the items; thus, higher scores indicated higher levels of honesty-humility. Internal consistency was high (α = .82).

**Altruism.** Participants similarly completed the altruism subscale of the HEXACO Personality Inventory [1], which consisted of four items (e.g., “I try to give generously to those in need”) that participants rated their agreement with on a 5-point scale (1 = *Strongly disagree* to 5 = *Strongly agree*). This was scored by averaging all responses to the items; thus, higher scores indicated higher levels of altruism. Internal consistency was low (α = .56).

**Results**

**Descriptive statistics**

Bivariate correlations and descriptive statistics for all variables are included in S1 Table. Women reported higher honesty-humility, *t*(302) = -3.74, *p* < .001, *d* = -0.45, and altruism scores, *t*(302) = -4.64, *p* < .001, *d* = -0.56, than did men.

| **S1 Table. Descriptive Statistics and Correlations among Variables in Study 1.** | | | | | | | | | |
| --- | --- | --- | --- | --- | --- | --- | --- | --- | --- |
|  | Variable | 1 | 2 | 3 | 4 | 5 | 6 | *M* | *SD* |
| (1) | Perceived Partner Commitment |  | -.21^**^ | .27^**^ | .45^**^ | -.11 | .03 | 7.65 | 1.40 |
| (2) | Agreeableness | -.22^*^ |  | -.40^**^ | -.32^**^ | -.16^*^ | -.21^**^ | 3.25 | 0.41 |
| (3) | Honesty-Humility | .36^**^ | -.36^**^ |  | -.48^**^ | -.12 | -.05 | 3.49 | 0.65 |
| (4) | Altruism | .50^**^ | -.30^**^ | .55^**^ |  | -.14 | .03 | 4.09 | 0.70 |
| (5) | Selfishness Questionnaire | -.15 | -.11 | -.32^**^ | -.30^**^ |  | .35^**^ | 1.55 | 0.44 |
| (6) | Welfare Tradeoff | -.13 | -.03 | -.23^*^ | -.12 | .38^**^ |  | 0.07 | 0.96 |
|  | *M* | 7.20 | 3.30 | 3.21 | 3.69 | 1.75 | -0.10 |  |  |
|  | *SD* | 1.62 | 0.43 | 0.62 | 0.77 | 0.52 | 1.07 |  |  |
| *Note.* Descriptive statistics and correlations are presented above the diagonal for women and below the diagonal for men.  ^*^ *p* < .05. ^**^ *p* < .01. | | | | | | | | | |

**Do** **similar variables also moderate the association between perceived partner commitment and selfishness?**

To assess whether similar variables also determine the implications of perceived partner commitment, we conducted two supplemental sets of analyses. The first addressed the role of honesty-humility. The first model addressing honesty-humility regressed participants’ scores on the *welfare trade-off task* onto mean-centered perceived partner commitment scores, mean-centered honesty-humility scores, and their interaction. Results of these analyses are presented in the left columns of S2 Table. As shown, the Perceived Partner Commitment × Honesty-Humility interaction significantly predicted scores on the welfare trade-off task (S1 Fig), but tests of the simple slopes revealed perceived partner commitment did not predict welfare trade-off scores among participants one standard deviation above, *b* = 0.08, *SE* = 0.06, *t*(300) = 1.44, *p* = .152, *r* = .08, or below the mean, *b* = -0.06, *SE* = 0.05, *t*(300) = -1.19, *p* = .234, *r* = -.07, in honesty-humility. The second model addressing honesty-humility regressed participants’ scores on the *selfishness questionnaire* onto mean-centered perceived partner commitment scores, mean-centered honesty-humility scores, and their interaction. Results of these analyses are presented in the right columns of S2 Table. As shown, honesty-humility did have a significant positive main effect on self-reported selfishness. However, this main effect was qualified by a significant Perceived Partner Commitment × Honesty-Humility interaction (S2 Fig). Tests of the simple slopes revealed that perceived partner commitment was negatively associated with self-reported selfishness for people one standard deviation below the mean in honesty-humility, *b* = -0.07, *SE* = 0.02, *t*(303) = -2.98, *p* = .003, *r* = -.17, but not for people one standard deviation above the mean in honesty-humility, *b* = 0.02, *SE* = 0.03, *t*(303) = 0.87, *p* = .384, *r* = .05.

| **S2 Table. Effects of Perceived Partner Commitment, Honesty-Humility, and Their Interaction on Selfishness in Study 1.** | | | | | | | | | |
| --- | --- | --- | --- | --- | --- | --- | --- | --- | --- |
|  | Welfare Trade-Off | | | | Selfishness Questionnaire | | | | |
| Measure | *b* | *t* | *r* | *p* | | *b* | *t* | *r* | *p* |
| PPC | 0.01 | 0.27 | .02 | .790 | | -0.02 | -1.23 | -.07 | .221 |
| Honesty-Humility | -0.17 | -1.84 | -.11 | .067 | | -0.15 | -3.60 | -.20 | < .001 |
| PPC × Honesty-Humility | 0.11 | 2.05 | .12 | .041 | | 0.07 | 2.86 | .16 | .005 |
| *Note.* PPC = Perceived Partner Commitment. For the welfare trade-off task, *df* = 300. For the selfishness questionnaire, *df* = 303. | | | | | | | | | |

**S1 Fig. Interactive Effects of Perceived Partner Commitment and Honesty-Humility on Selflessness, as Measured by the Welfare Trade-Off Task, in Study 1.**


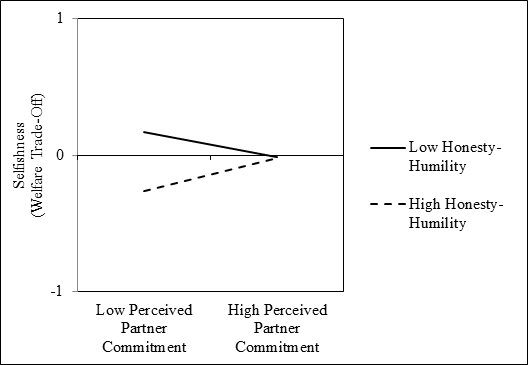


**S2 Fig. Interactive Effects of Perceived Partner Commitment and Honesty-Humility on Selflessness, as Measured by the Selfishness Questionnaire, in Study 1.**


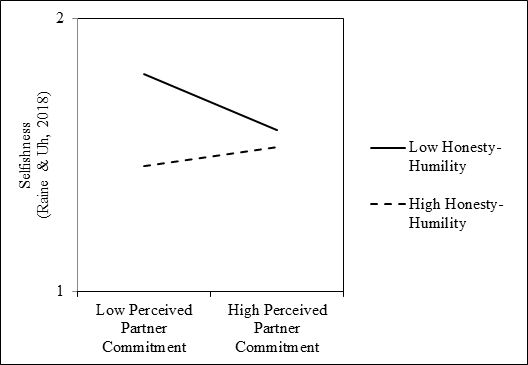


The second set of supplemental analyses addressed the role of altruism. The first model addressing altruism regressed participants’ scores on the *welfare trade-off task* onto mean-centered perceived partner commitment scores, mean-centered altruism scores, and their interaction. Results of these analyses are presented in the left columns of S3 Table. As shown, altruism did not have a significant main effect on welfare trade-off scores, nor was there a significant interaction between perceived partner commitment and altruism on welfare trade-off scores. The second model addressing altruism regressed participants’ scores on the *selfishness questionnaire* onto mean-centered perceived partner commitment scores, mean-centered altruism scores, and their interaction. Results of these analyses are presented in the right columns of S3 Table. As shown, there was a significant main effect of altruism on self-reported selfishness. However, this main effect was qualified by a significant Perceived Partner Commitment × Altruism interaction (S3 Fig). Tests of the simple slopes revealed perceived partner commitment was positively associated with self-reported selfishness for participants one standard deviation above the mean in altruism, *b* = 0.07, *SE* = 0.03, *t*(303) = 2.32, *p* = .021, *r* = .13, but negatively associated with self-reported selfishness for participants one standard deviation below the mean in altruism, *b* = -0.08, *SE* = 0.03, *t*(303) = -3.00, *p* = .003, *r* = -.17.

| **S3 Table. Effects of Perceived Partner Commitment, Altruism, and Their Interaction on Selfishness in Study 1.** | | | | | | | | |
| --- | --- | --- | --- | --- | --- | --- | --- | --- |
|  | Welfare Trade-Off | | | | Selfishness Questionnaire | | | |
| Measure | *b* | *t* | *r* | *p* | *b* | *t* | *r* | *p* |
| PPC | -0.02 | -0.45 | -.03 | .654 | -0.00 | -0.19 | -.01 | .853 |
| Altruism | 0.02 | 0.18 | .01 | .856 | -0.13 | -3.21 | -.18 | .001 |
| PPC × Altruism | 0.04 | 0.76 | .04 | .448 | 0.10 | 3.87 | .22 | < .001 |
| *Note.* PPC = Perceived Partner Commitment. For the welfare trade-off task, *df* = 300. For the selfishness questionnaire, *df* = 303. | | | | | | | | |

**S3 Fig. Interactive Effects of Perceived Partner Commitment and Altruism on Selflessness, as measured by the Selfishness Questionnaire, in Study 1.**


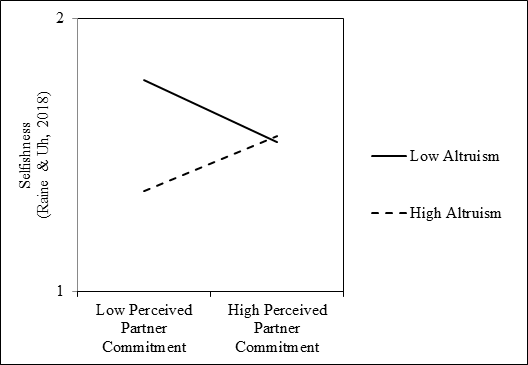


**Study 3**

**Method**

**Materials**

**Honesty-Humility.** To rule out other potential moderators, we included the honesty-humility scale [1] described in Study 1. This questionnaire was scored by averaging all responses to the items; thus, higher scores indicated higher levels of honesty-humility. Internal consistency was acceptable (α = .76).

**Dark Tetrad Traits.** We similarly included the short Dark Tetrad Scale [2] that consisted of 28 items that participants indicated their agreement with on a 5-point scale (1 = *Strongly disagree* to 5 = *Strongly agree*). The original version of this scale uses four subscales: narcissism (e.g., “I like to show off every now and then”), Machiavellianism (“e.g., “Keep a low profile if you want to get your way”), psychopathy (e.g., “People often say I’m out of control”), and sadism (e.g., “Some people deserve to suffer”). We created both a total scale score by averaging all responses to the items and individual subscale scores by averaging all responses to the items that correspond to each trait; thus, higher scores indicated higher levels of the Dark Tetrad traits for both the total scale score and individual subscale scores. Internal consistency was high (total scale, α = .84; narcissism, α = .70, Machiavellianism, α = .66; psychopathy, α = .70; sadism, α = .80).

**Results**

**Descriptive statistics**

Bivariate correlations and descriptive statistics are included in S4 Table. As shown, and consistent with Study 1, participants reported honesty-humility scores above the midpoint, suggesting that they saw themselves as fair and genuine, on average. Men and women did not differ significantly on agreeableness, *t*(247) = -1.32, *p* = .094, *d* = -0.17, or honesty-humility scores, *t*(247) = -0.71, *p* = .240, *d* = -0.09. In addition, participants reported Dark Tetrad scores around the midpoint, with males reporting higher Dark Tetrad scores than females, *t*(247) = 4.70, *p* < .001, *d* = 0.60.

| **S4 Table. Descriptive Statistics and Correlations among Variables in Study 3** | | | | | | | | | | | | |
| --- | --- | --- | --- | --- | --- | --- | --- | --- | --- | --- | --- | --- |
|  | Variable | 1 | 2 | 3 | 3.1 | 3.2 | 3.3 | 3.4 | 4 | 5 | *M* | *SD* |
| (1) | Agreeableness | **.14** | .38^**^ | -.34^**^ | -.07 | -.24^**^ | -.40^**^ | -.26^**^ | -.28^**^ | .09 | 3.89 | 0.42 |
| (2) | Honesty-Humility | .51^**^ | **.15** | -.45^**^ | -.20^*^ | -.50^**^ | -.32^**^ | -.29^**^ | -.26^**^ | .07 | 3.59 | 0.52 |
| (3) | Dark Tetrad Total Scale | -.53^**^ | -.62^**^ | **.10** | .66^**^ | .63^**^ | .78^**^ | .75^**^ | .21^*^ | -.09 | 2.53 | 0.47 |
|  | (3.1) Narcissism | -.17 | -.33^**^ | .55^**^ | **.15** | .26^**^ | .38^**^ | .22^**^ | .04 | -.18^*^ | 3.07 | 0.69 |
|  | (3.2) Machiavellianism | -.34^**^ | -.41^**^ | .66^**^ | .15 | **.12** | .28^**^ | .28^**^ | .12 | .01 | 3.13 | 0.62 |
|  | (3.3) Psychopathy | -.50^**^ | -.49^**^ | .70^**^ | .24^*^ | .33^**^ | **.10** | .56^**^ | .26^**^ | -.02 | 1.81 | 0.63 |
|  | (3.4) Sadism | -.42^**^ | -.46^**^ | .79^**^ | .21^*^ | .38^**^ | .38^**^ | **.05** | .17^*^ | -.06 | 2.12 | 0.73 |
| (4) | Selfishness Questionnaire | -.41^**^ | -.38^**^ | .29^**^ | .09 | .29^**^ | .14 | .25^**^ | **.04** | .28^**^ | 1.27 | 0.28 |
| (5) | Noise Blast Volume Choice | -.03 | -.02 | .12 | .15 | .16 | -.09 | .10 | .16 | **.04** | 46.63 | 22.39 |
|  | *M* | 3.82 | 3.55 | 2.82 | 3.05 | 3.26 | 2.10 | 2.85 | 1.26 | 33.78 |  |  |
|  | *SD* | 0.43 | 0.53 | 0.47 | 0.61 | 0.61 | 0.63 | 0.88 | 0.24 | 23.36 |  |  |
| *Note.* Descriptive statistics and correlations are presented above the diagonal for women and below the diagonal for men; correlations between partners appear on the diagonal in bold.  ^*^ *p* < .05. ^**^ *p* < .01. | | | | | | | | | | | | |

**Do** **similar variables also moderate the association between perceived partner commitment and selfishness?**

To assess whether similar variables also determine the implications of perceived partner commitment, we conducted two supplemental sets of analyses. The first addressed the role of honesty-humility. The first model assessing honesty-humility examined the implications of perceived partner commitment for the *noise blast task* by estimating a two-level model that regressed participants’ volume choice onto condition, mean-centered honesty-humility scores, and their interaction. Results of these analyses are presented in the left columns of S5 Table. As shown, honesty-humility did not predict volume choice and the PPC Condition × Honesty-Humility interaction did not significantly predict volume choice. The second model addressing honesty-humility examined the implications of perceived partner commitment for participants’ *self-reported selfishness* by regressing participants’ scores on the selfishness questionnaire onto condition, mean-centered honesty-humility scores, and their interaction. Results of these analyses are presented in the right columns of S5 Table. As shown, honesty-humility did predict self-reported selfishness, but, similar to the noise blast task, the PPC Condition × Honesty-Humility interaction did not significantly predict self-reported selfishness.

| **S5 Table. Effects of Perceived Partner Commitment Condition, Honesty-Humility, and Their Interaction on Selfishness in Study 3** | | | | | | | | |
| --- | --- | --- | --- | --- | --- | --- | --- | --- |
|  | Volume Choice (Noise Blast Task) | | | | Selfishness Questionnaire | | | |
| Measure | *b* | *t* | *r* | *p* | *b* | *t* | *r* | *p* |
| PPC Condition | -0.20 | -0.13 | -.01 | .894 | -0.00 | -0.07 | -.01 | .943 |
| Honesty-Humility | 1.92 | 0.69 | .06 | .493 | -0.15 | -5.21 | -.43 | <.001 |
| PPC × Honesty-Humility | -5.28 | -1.81 | -.16 | .073 | -0.05 | -1.61 | -.15 | .110 |
| *Note.* PPC = Perceived Partner Commitment. For the noise blast task, *df* = 120. For the selfishness questionnaire, *df* = 120. | | | | | | | | |

The second set of supplemental analyses addressed the role of the Dark Tetrad traits. First, we chose to treat the Dark Tetrad measure as a total score of all four traits. The first model addressing the Dark Tetrad examined the implications of perceived partner commitment for the *noise blast task* by estimating a two-level model that regressed participants’ volume choice onto condition, mean-centered Dark Tetrad scores, and their interaction. Results of these analyses are presented in the left columns of S6 Table. As shown, Dark Tetrad scores did not significantly predict volume choice, and the PPC Condition × Dark Tetrad interaction did not significantly predict volume choice. The second model addressing the Dark Tetrad examined the implications of perceived partner commitment for participants’ *self-reported selfishness* by regressing participants’ scores on the selfishness questionnaire onto condition, mean-centered Dark Tetrad scores, and their interaction. Results of these analyses are presented in the right columns of S6 Table. As shown, Dark Tetrad scores were significantly positively associated with self-reported selfishness. However, the PPC Condition × Dark Tetrad interaction did not significantly predict self-reported selfishness.

| **Table 6-OSM**  *Effects of Perceived Partner Commitment Condition, Dark Tetrad Traits, and Their Interactions on Selfishness in Study 3* | | | | | | | | | | |
| --- | --- | --- | --- | --- | --- | --- | --- | --- | --- | --- |
|  | Volume Choice  (Noise Blast Task) | | | | Selfishness  Questionnaire | | | | | |
| Measure | *b* | *t* | *r* | *p* | | *b* | *t* | *r* | *p* |  |
| *Composite DT* | | | | | | | | | |  |
| PPC Condition | -0.23 | -0.15 | -.01 | .881 | | -0.00 | -0.05 | -.00 | .962 |  |
| Dark Tetrad | -4.81 | -1.61 | -.15 | .111 | | 0.12 | 2.93 | .26 | .004 |  |
| PPC × Dark Tetrad | 3.98 | 1.29 | .12 | .200 | | 0.02 | 0.45 | .04 | .654 |  |
| *Narcissism* | | | | | | | | | |  |
| PPC Condition | -0.16 | -0.10 | -.01 | .918 | | -0.00 | -0.17 | -.02 | .866 |  |
| Narcissism | -1.20 | -0.52 | -.05 | .605 | | 0.02 | 0.81 | .07 | .418 |  |
| PPC × Narcissism | -0.33 | -0.15 | -.01 | .885 | | -0.00 | -0.16 | -.01 | .872 |  |
| *Machiavellianism* | | | | | | | | | |  |
| PPC Condition | -0.12 | -0.08 | -.01 | .940 | | 0.00 | 0.00 | .00 | .999 |  |
| Machiavellianism | 1.39 | 0.55 | .05 | .584 | | 0.08 | 2.40 | .21 | .018 |  |
| PPC × Machiavellianism | 3.31 | 1.26 | .11 | .211 | | 0.03 | 1.05 | .10 | .297 |  |
| *Psychopathy* | | | | | | | | | |  |
| PPC Condition | -0.16 | -0.11 | -.01 | .916 | | -0.00 | -0.14 | -.01 | .886 |  |
| Psychopathy | -5.24 | -2.39 | -.21 | .019 | | 0.08 | 2.70 | .24 | .008 |  |
| PPC × Psychopathy | 4.93 | 2.27 | .20 | .025 | | 0.01 | 0.44 | .04 | .665 |  |
| *Sadism* | | | | | | | | | |  |
| PPC Condition | -0.15 | -0.10 | -.01 | .920 | | -0.00 | -0.16 | -.01 | .871 |  |
| Sadism | -3.37 | -1.99 | -.18 | .049 | | 0.05 | 2.95 | .26 | .004 |  |
| PPC × Sadism | 1.16 | 0.64 | .06 | .523 | | -0.01 | -0.31 | -.03 | .754 |  |
| *Note.* DT = Dark Tetrad. PPC = Perceived Partner Commitment. For the noise blast task, *df* = 120. For the selfishness questionnaire, *df* = 120. | | | | | | | | | | |

To further break down potential associations between the Dark Tetrad traits and selfishness, we ran two models (one for each operationalization of selfishness) for each Dark Tetrad trait. The first models examined the implications of perceived partner commitment for the *noise blast task* by estimating four two-level models that regressed participants’ volume choice onto condition, mean-centered subscale scores (narcissism, Machiavellianism, psychopathy, and sadism), and their interactions. Results of these analyses are presented in the left columns of S6 Table. As shown, sadism and psychopathy were significantly negatively associated with volume choice. However, the PPC Condition × Psychopathy interaction was significant (S4 Fig), but tests of the simple slopes revealed that perceived partner commitment did not predict volume choice for participants one standard deviation above the mean, *b* = 3.04, *SE* = 2.04, *t*(120) = 1.49, *p* = .139, *r* = .13, and below the mean in psychopathy, *b* = -3.36, *SE* = 2.10, *t*(120) = -1.60, *p* = .112, *r* = -.14. There were no significant main effects for narcissism or Machiavellianism, and there were no significant interactions for narcissism, Machiavellianism, or sadism. The second models examined the implications of perceived partner commitment for participants’ *self-reported selfishness* by regressing participants’ scores on the selfishness questionnaire onto condition, mean-centered subscale scores (narcissism, Machiavellianism, psychopathy, and sadism), and their interaction. Results of these analyses are presented in the right columns of Table 5-OSM. As shown, Machiavellianism, psychopathy, and sadism were significantly positively associated with self-reported selfishness. However, there were no significant interactions for narcissism, Machiavellianism, psychopathy, or sadism for self-reported selfishness.

**S4 Fig. Interactive Effects of Perceived Partner Commitment and Psychopathy on Selfishness, as measured by the Noise Blast Task, in Study 3.**


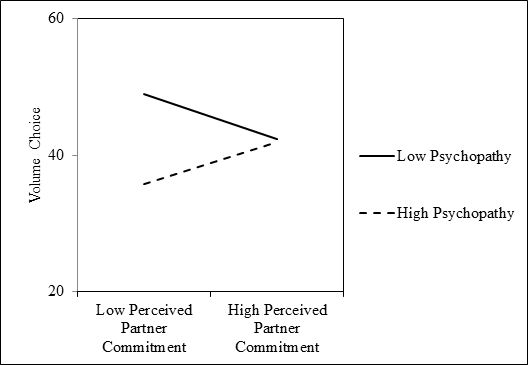


**References**

1. Lee K, Ashton MC. Psychometric properties of the HEXACO-100. Assessment. 2018 Jul;25(5):543-56.
2. Paulhus DL, Buckels EE, Trapnell PD, Jones DN. Screening for dark personalities. European Journal of Psychological Assessment. 2020 Jul 27.
